# Secondary Bacterial Infections in Patients with Seasonal Influenza A and Pandemic H1N1

**DOI:** 10.1155/2013/376219

**Published:** 2013-06-20

**Authors:** Karin Liderot, Marcus Ahl, Volkan Özenci

**Affiliations:** ^1^Division of Clinical Microbiology F 72, Karolinska Institutet, Karolinska University Hospital, Huddinge, 141 86 Stockholm, Sweden; ^2^Department of Infectious Diseases, Karolinska University Hospital, Huddinge, 141 86 Stockholm, Sweden

## Abstract

The aim of the present study is to analyse the secondary bacterial infections in a large group of patients with seasonal influenza A and influenza A(H1N1) pdm09. Patients diagnosed with seasonal influenza A and influenza A(H1N1) pdm09 between 2005 and 2009 were enrolled in the study. Data was retrieved from medical records and laboratory information systems (LIS). In total, 1094 patients with laboratory confirmed influenza were studied. There were 352 patients with seasonal influenza A and 742 patients with influenza A(H1N1) pdm09. The patients with influenza A were older and had higher comorbidity than patients with influenza A(H1N1) pdm09 (*P* < 0.001 and *P* < 0.05, resp.). Hospital admission was higher in influenza A group (*P* = 0.01). In contrast, ICU admission was higher in patients with influenza A(H1N1) pdm09 than influenza A patients (*P* < 0.05). There were higher numbers of bacterial samples taken and culture positivity in patients with influenza A than patients with influenza A(H1N1) pdm09 (*P* < 0.0001 and *P* = 0.01, resp.). In both groups, the majority of the patients with positive bacterial cultures had underlying diseases. The present study shows that the patient characteristics and the frequency of secondary bacterial infections were different in patients with seasonal influenza A and in patients with influenza A(H1N1) pdm09.

## 1. Introduction

The interaction between human influenza viruses with different subtypes of human and animal influenza viruses has been shown to give rise to new variants of the virus with pandemic potential [1–3]. All three previous pandemics, 1918, 1957, and 1968, contributed to excess mortality partly due to secondary bacterial infections [4]. In the case of the 1918 pandemic, mortality rates as high as 2.5% were reported. It was suggested that the secondary bacterial infections were the underlying reason for these excessive mortality rates [5]. After the 1968 pandemic, seasonal epidemics of influenza virus were dominated by A/H3N2 virus variants generated by antigenic drift [1, 5], and no new pandemics occurred during this time. However, in April 2009, a new influenza A/H1N1 virus emerged among humans in Mexico and California, quickly spreading worldwide between humans generating the first influenza pandemic of the 21st century [6].

During previous influenza pandemics, secondary bacterial infections caused by *Streptococcus pneumoniae, Haemophilus influenzae, Staphylococcus aureus*, and *Streptococcus pyogenes* have been important contributors to morbidity and mortality [4, 7, 8]. The information on the epidemiology of secondary bacterial infections might, therefore, play a significant role in reducing mortality and morbidity rates due to influenza by early implementation of accurate empiric antibacterial therapy.

Previous studies have suggested that influenza A(H1N1) pdm09 differs from the previous influenza pandemics, and secondary bacterial infections seem to play a limited role in influenza deaths during the current pandemic [9, 10]. However, to our knowledge, there is no previous comparative study on secondary bacterial infections due to seasonal influenza A and influenza A(H1N1) pdm09. These types of comparative studies might help us to understand the characteristics of the secondary bacterial infections with respective influenza type. 

The aim of the present study is to analyse the secondary bacterial infections in a large group of patients with seasonal influenza A and influenza A(H1N1) pdm09. 

## 2. Methods

### 2.1. Study Material and Design

A retrospective study analysing the presence of secondary bacterial infections in patients with either seasonal influenza A (between 2005 and 2008) or influenza A(H1N1) pdm09 (2009) was designed. All patients diagnosed with seasonal influenza A and influenza A(H1N1) pdm09 between 2005 and 2009 were enrolled in the study. Patients with influenza between 2005 and 2008 were grouped as “influenza A”. It is important to note that patients in the “influenza A” group were not homogeneous since several different influenza viruses including H1N1 and H3N2 dominated during different periods of time between 2005 and 2008. It was not possible to subgroup these patients according to the type of influenza virus since the molecular typing of respective virus for each patient was not performed. The patients that had positive bacteriological findings in both groups were studied further. Reviewers used a standardized form. All data was abstracted from medical records including age, sex, presence of comorbid conditions, and clinical presentation and course including autopsy reports, length of hospital stay (including intensive care unit stay), and use of antibiotic/antiviral treatment. A positive bacteriological finding was defined as any growth in blood cultures or growth of relevant pathogenic airway bacteria in airway samples. The microbiology results were collected from medical records and laboratory information systems (LIS). 

### 2.2. Detection of Virus

Flocked swabs or nasopharyngeal aspirates had been used to collect respiratory epithelial cells from the posterior nasopharynx. 

The laboratory diagnosis of seasonal influenza A was made by detection of influenza A-antigen with immunofluorescence (IF). Samples were centrifuged at 1000 rpm for 10 min to pellet the cells for direct immunofluorescence (DFA). The cell pellets were then resuspended in a small amount of phosphate-buffered saline, and 100 *μ*L was applied to glass slides, by cytocentrifugation (Shandon Cytospin 2, Thermo Scientific, Waltham, MA, USA) at 1200 rpm for 10 min. The slides were air-dried and then fixed in cold acetone for 10 min. The cell spots were stained with 25 *μ*L of conjugated monoclonal antibodies from a commercial DFA kit from PathoDx Respiratory Virus Panel (Diagnostic Product Corporation, Los Angeles, CA, USA) for 15 minutes at 37°C. Evan's Blue was used as a counterstain. After repeated washing in phosphate-buffered saline, the slides were mounted in 70% glycerol and examined under microscope. A positive result was indicated by the presence of at least one intact cell showing specific fluorescence using a fluorescence microscope (Carl Zeiss, Oberkochen, Germany). The presence of ≥50 epithelial cells per glass slide was required for the specimen to be considered adequate for DFA testing. Positive and negative controls from PathoDx as well as controls from cultivated influenza A strains were used.

For diagnosing pandemic influenza H1N1, real-time-reverse transcriptase-polymerase chain reaction (rRT-PCR) with subtype-specific primers for influenza A(H1N1) pdm09 was used. A sample volume of 400 *μ*L was extracted using the MagAttract Virus Mini M48 Kit (Qiagen GmbH, Hilden, Germany) according to the manufacturer's instructions with the Qiagen M48 BioRobot. The RNA was eluted to a final volume of 100 *μ*L. Water was used as a negative control and an influenza virus isolate, A/Stockholm2/2009 H1N1, was used as a positive control for each extraction. The PCR method used was a one-step rRT-PCR provided by the Swedish Institute for Communicable Disease Control. The primers used were 5′ GGC TGC TTT GAA TTT TAC CAC AA 3′ and 5′ TTT GGG TAG TCA TAA GTC CCA TTT T 3′, amplifying the haemagglutinin gene. The probe used was 5′-FAM-TGC GAT AAC ACG TGC ATG GAA AGT GTC-TAMRA-3′. For the PCR, the SuperScript III Platinum One-Step Quantitative RT-PCR system was used (Invitrogen Corporation, Carlsbad, CA, USA). The PCR program used was reverse transcription for 15 minutes at 50°C followed by 2 minutes at 95°C, 45 cycles of 95°C for 5 seconds, 60°C for 60 seconds, and 40°C for 30 seconds using LightCycler 480 (Roche Diagnostics GmbH, Mannheim, Germany). A threshold cycle (*C*_*T*_) of ≤40 together with a sigmoid fluorescence curve was needed for the result to be considered positive.

### 2.3. Detection of Bacteria

All patients with positive seasonal influenza A or influenza A(H1N1) pdm09 were cross-examined in the clinical microbiology laboratory database of Karolinska University Hospital, Huddinge, for the presence of positive bacteria cultures from airways or blood. Blood samples were cultured in the BacT/ALERT 3D (bioMérieux Inc., Durham, NC, USA) automated blood culture system. Detection of bacteria from clinical samples was made by standard methods. 

## 3. Statistical Analysis

The Fisher exact test and Students *t*-test were used in categorical comparison and in comparing the two groups, respectively. Values of *P* < 0.05 were considered as statistically significant. 

### 3.1. Ethical Permission

The study was approved by the Regional Ethical Review Board of Stockholm, Sweden (Dnr 2010/266-31). 

## 4. Results

### 4.1. Bacterial Findings

In total, 1094 patients with laboratory confirmed influenza were studied. In the study group, there were 352 patients with seasonal influenza A and 742 patients with influenza A(H1N1) pdm09. The numbers of bacteriological sampling were higher in patients with seasonal influenza A than patients with influenza A(H1N1) pdm09, 240/352 (68.18%) versus 99/742 (13.34%), respectively (*P* < 0.0001). 

The positive bacterial cultures were analysed further in relation to total numbers of influenza A(H1N1) pdm09 and influenza A patients. Among the influenza A patients, 33/352 (9.38%) had positive bacteriological samples. The numbers of influenza A positive patients who had positive cultures from upper airways, lower airways, and blood were 20/352 (5.68%), 13/352 (3.69%), and 8/352 (2.27%), respectively (Table 1).

In 38/742 (5.12%) of the influenza A(H1N1) pdm09 positive patients, the cultures were positive for bacterial growth. The numbers of influenza A(H1N1) pdm09 positive patients who had positive cultures from upper airways, lower airways, and blood were 15/742 (2.02%), 12/742 (1.62%), and 12/742 (1.62%), respectively. The different bacterial species identified in cultures are depicted in Table 1. There were no cases with methicillin-resistant *Staphylococcus aureus* (MRSA) in the studied material.

In total, patients with influenza A had higher numbers of positive cultures than patients with influenza A(H1N1) pdm09 (*P* = 0.01). Numbers of positive upper- and lower-airway cultures were elevated in patients with influenza A than patients with influenza A(H1N1) pdm09 (*P* < 0.01 and *P* < 0.05, resp.). No difference was observed in the numbers of positive blood cultures between the two groups (Table 1).

### 4.2. Patient Characteristics

In order to understand the possible underlying mechanisms for the occurrence of secondary bacterial infections, the characteristics of patients with positive relevant bacteriological findings in airways and blood were analysed in detail. The medical records of these patients were reviewed and the baseline characteristics are depicted in Table 2. For these patients, the clinical significance of the bacterial findings was determined by assessment of the clinicians' response to the results from the microbiology laboratory.

There were differences in baseline characteristics between patients with seasonal influenza A and patients with influenza A(H1N1) pdm09. The median age of patients with seasonal influenza A group was significantly higher than influenza A(H1N1) pdm09 group, 57.5 years versus 30.5 years, respectively (*P* ≤ 0.001). Women were the majority in both patient groups, 57.58% in seasonal influenza A patients and 65.79% among influenza A(H1N1) pdm09 patients.

Total numbers of patients with comorbidity were significantly higher in the seasonal influenza A group (*P* < 0.05). When comparing prestudy morbidity in the groups, the patients with seasonal influenza A had significantly more cardiovascular disease than the influenza A(H1N1) pdm09 patients 7/33 (21.21%) versus 1/38 (2.63%), respectively (*P* < 0.05). Two out of 33 (6%) seasonal influenza A patients had diabetes mellitus, compared to none among the influenza A(H1N1) pdm09 patients. Conditions leading to immunosuppression were equally common in the two groups and included lymphoproliferative disease, acute myeloid leukemia (AML) or chronic lymphocytic leukemia (CLL), stem cell transplantation, and secondary immunodeficiency (hypogammaglobulinemia or HIV) (data not shown). The presence of chronic lung disease and chronic renal disease was similar in both groups. 

Median time between onset of symptoms and time to seeking medical care was similar in both study groups. In contrast, there were considerable differences in length of hospital stay and intensive care unit (ICU) admission. Influenza A patients were more often admitted to hospital than influenza A(H1N1) pdm09 patients 30/33 (90.90%) versus 24/38 (63.16%), respectively (*P* = 0.01). Despite high hospital admission rates among the influenza A patients, the duration of the hospital stay was shorter in the seasonal influenza A patients compared to influenza A(H1N1) pdm09 patients, 8.5 versus 13.5 days, respectively, although this difference was not statistically significant (*P* = 0.06). Interestingly, ICU admittance was lower in seasonal influenza A patients compared to influenza A(H1N1) pdm09 patients, 2/33 (6.06%) versus 11/38 (28.95%), respectively (*P* < 0.05). 

### 4.3. Antimicrobial Treatment

Antiviral treatment (Oseltamivir or Zanamivir) was used in 10/33 (30.30%) patients with seasonal influenza A and in 18/38 (47.37%) patients with influenza A(H1N1) pdm09. Treatment time was 5 days for all seasonal influenza A patients and most of the influenza A(H1N1) pdm09 patients. Extended antiviral treatment was given to 6 patients with influenza A(H1N1) pdm09 that had a long ICU stay (>7 days). Length of antiviral treatment in these patients ranged between 6 and 35 days (mean = 22 days). All of these patients suffered from preexisting hematological disease (myeloma, CLL, or AML) or severe chronic lung disease. Several of these patients remained PCR positive for influenza A(H1N1) pdm09 throughout their ICU stay, despite antiviral treatment.

Numbers of seasonal influenza A patients and influenza A(H1N1) pdm09 patients who received antibacterial treatment were 26/33 (78.79%) and 25/38 (65.79%), respectively (Table 2). There was a greater heterogeneity in which antibacterial regime that was chosen in the seasonal influenza A epidemics. Penicillin and amoxicillin were most commonly used as oral (PO) therapy, closely followed by doxycycline. In more severe cases, when intravenous (IV) administration was preferred, cefuroxime alone or penicillin G combined with an aminoglycoside was chosen as empiric therapy. When a switch from IV to PO therapy was made, amoxicillin or doxycycline was used. Other empiric antibiotic choices were carbapenems, quinolones, and macrolides combined with aminoglycoside. Antibiotic treatments typically lasted for 7–10 days. Patients treated in the ICU for a long time (>7 days) received IV antimicrobial therapy with a broader spectrum. This was, in the majority of cases, related to ventilator-associated airway infections or other ICU-related infections (data not shown).

When influenza A(H1N1) pdm09 patients were treated with PO antibiotics, amoxicillin or amoxicillin-clavulanic acid was preferred as empiric therapy. When IV therapy was chosen, penicillin G or a second/third-generation cephalosporin was most commonly used. Patients admitted to the ICU always received a second/third-generation cephalosporin. In cases with severe inflammatory response and/or severe respiratory failure, a quinolone (moxifloxacin) was added as empiric therapy. In cases of septic shock/ARDS, cephalosporin therapy was often combined with aminoglycosides. Piperacillin/tazobactam and carbapenems were rarely used in the ICU patients unless they were treated in the ICU for a long time (>7 days) (data not shown). 

## 5. Discussion

Secondary bacterial infections have been important contributors to morbidity and mortality during the previous influenza pandemics [4, 7, 11]. It has been shown that *S. pneumoniae* and *S. aureus* have contributed to excess mortality in these patient groups [12]. There is no previous study comparing the characteristics and the occurrence of secondary bacterial infections with different types of influenza viruses in the same setting. 

Here, we analysed the secondary bacterial infections in a large group of patients with seasonal influenza A and influenza A(H1N1) pdm09 influenza, diagnosed at Karolinska University Hospital, during 2005–2009. 

The numbers of influenza A(H1N1) pdm09 positive patients in one year were significantly higher than the numbers of patients with seasonal influenza A in 4 years, 742 versus 352 patients, respectively. During 2009, the diagnostic tests in order to identify influenza A(H1N1) pdm09 were used extensively. The clinicians were instructed by the Swedish National Board of Health and Welfare to test every suspected case of influenza A(H1N1) pdm09 influenza to keep track of the extension of the pandemic and to identify the patients in the risk groups that should receive antiviral treatment. The high numbers of influenza A(H1N1) pdm09 positive patients in the study might probably depend on high numbers of sampling for this virus. In the study, the diagnostic methods used for seasonal influenza A and influenza A(H1N1) pdm09 were also different. The PCR method which is used for influenza A(H1N1) pdm09 has previously shown to be more sensitive than IF, which was used for seasonal influenza A between 2005 and 2008 [13]. The difference in the performance of the two methods might also play a role in high numbers of influenza A(H1N1) pdm09 positivity. Another possibility is that the influenza A(H1N1) pdm09 virus infected higher numbers of patients than seasonal influenza A virus. 

Previous studies have shown that influenza A(H1N1) pdm09 influenza mostly infects young patients [14, 15]. The present data suggest that the young influenza A(H1N1) pdm09 infected patients who develop severe influenza often suffer from preexisting complicating diseases. Hospital admission was higher in influenza A group (*P* = 0.01). In contrast, ICU admission was higher in patients with influenza A(H1N1) pdm09 than influenza A patients (*P* < 0.05). The influenza A(H1N1) pdm09 patients, as a group being younger and healthier, were probably more likely to be discharged from the emergency room, but the individuals that were hospitalized often had a more severe course of infection, especially in the subgroup of individuals suffering from immunosuppression.

There were higher numbers of bacterial samples taken and culture positivity in patients with influenza A than patients with influenza A(H1N1) pdm09 (*P* < 0.0001 and *P* = 0.01, resp.). However, the two patient groups in the study differed in size and partly in selection since bacterial cultures were performed in a larger proportion in the seasonal influenza A patients. In both groups, a majority of the patients with positive bacterial cultures had underlying diseases. In blood cultures, *S. pneumoniae * and coagulase-negative *Staphylococcus* were the most common isolates in both groups. The three most common bacterial species isolated from lower airway samples in influenza A patients were *S. pneumoniae, S. aureus,* and *H. influenzae*. In influenza A(H1N1) pdm09 patients, lower airway cultures were instead dominated by *S. aureus, Streptococcus dysgalactiae,* and coagulase-negative staphylococci. The reason for this difference is not known.

In the studied material, growth of *S. aureus* and *S. pneumoniae* in lower airways or blood was shown to lead to antibiotic treatment. A majority of positive blood cultures with coagulase-negative staphylococci were interpreted as contamination and subsequently not subjected to antibiotic treatment. Patients with positive upper airway cultures received antibiotic treatment to a lesser extent than those who had a positive lower airway culture and blood culture. This could partly be explained by increased colonization of upper respiratory airways of many bacteria, including pathogens during acute viral respiratory disease, as shown by others previously. 

In the present study, 30.30% of the patients with seasonal influenza A and 47.37% of the influenza A(H1N1) pdm09 patients received antiviral treatment. During the pandemic, the Swedish National Board of Health and Welfare instructed physicians to initiate antiviral treatment within 2-3 days after the beginning of influenza symptoms. It has been shown that patients with influenza benefit from antiviral treatment if it is initiated within the first 4 days of illness [16]. Several patients with influenza A(H1N1) pdm09 sought medical help after 4 days of flu symptoms and, therefore, were disqualified for antiviral treatment. 

Despite a low frequency of secondary bacterial infections in the present and previous studies, a majority of the patients with either seasonal influenza A or influenza A(H1N1) pdm09 received antibacterial treatment. Our results concerning bacterial etiology in these infections suggest that empiric antibiotic therapy in influenza A and influenza A(H1N1) pdm09 patients should be directed primarily against *S. pneumoniae *and* S. aureus*. In the Swedish setting there is, in general, no need for empiric coverage of MRSA. 

In retrospect, the course of the influenza A(H1N1) pdm09 in Sweden was not as severe as first suspected. The lack of a great number of secondary bacterial infections, as presented here, might be one of the underlying reasons for the benign course of the disease, especially when it is compared with the 1918 pandemic [7]. However, for a few individuals, infections with the influenza A(H1N1) pdm09 had a severe course due to viral pneumonia as well as secondary bacterial infections. Therefore, early accurate diagnosis of both influenza as well as secondary bacterial infections might be important in reducing mortality and morbidity rates in these patients.

## Figures and Tables

**Table 1:** Numbers of patients with seasonal influenza A and pandemic H1N1 and numbers of relevant bacteria spp. isolated from these patients. Number (% of the total).

	Influenza A	H1N1	Statistical analysis
Total number of patients	352	742	
Year	2005–2008	2009	
Patients with bacterial samples taken	240 (68.18%)	99 (13.34%)	*P* < 0.0001
Patients with positive bacterial findings	33 (9.38%)	38 (5.12%)	*P* = 0.01
Respiratory cultures			
Patients with positive upper airway cultures*(nasopharynx/throat)	20 (5.68%)	15 (2.02%)	*P* < 0.01
* S. pneumoniae *	10 (2.84%)	10 (1.35%)	ND
* H. influenzae *	5 (1.42%)	4 (0.54%)	ND
* M. catarrhalis *	8 (2.27%)	3 (0.40%)	ND
* S. aureus *	0	3 (0.40%)	ND
* S. pyogenes (*group A streptococci)	2 (0.57%)	0	ND
Patients with positive lower airway cultures (sputum/bronchoalveolar lavage)	13 (3.69%)	12 (1.62%)	*P* < 0.05
* S. pneumoniae *	5 (1.42%)	(0.1%)	ND
* H. influenza *	2 (0.57%)	0	ND
* M. catarrhalis *	1 (0.28%)	1 (0.13%)	ND
* S. aureus *	3 (0.85%)	5 (0.67%)	ND
Coagulase negative staphylococci	0	2 (0.27%)	ND
* S. dysgalactiae (*group G streptococci)	0	2 (0.27%)	ND
* S. pyogenes (*group A streptococci)	1 (0.28%)	0	ND
* Enterobacteriaceae *	1 (0.28%)	1 (0.13%)	ND
Blood cultures**			
* *All positive blood cultures	8 (2.27%)	12 (1.62%)	NS
* S. pneumoniae *	1 (0.28%)	4 (0.54%)	ND
* S. aureus *	1 (0.28%)	1 (0.13%)	ND
* H. influenzae *	1 (0.28%)	0	ND
Viridans streptococci	1 (0.28%)	0	ND
* S. pyogenes (*group A streptococci)	0	1 (0.13%)	ND
* *Coagulase-negative staphylococci	4 (1.14%)	6 (0.81%)	ND

*Several patients had multiple findings. **One patient had multiple findings. ND: not determined; NS: not significant.

**Table 2:** Baseline characteristics of patients with seasonal influenza A and pandemic H1N1 with relevant positive bacteriological cultures. Number (% of the total).

	Influenza A	H1N1	Statistical analysis
Influenza patients with bacteriological relevant findings	33	38	
Age—median (min-max)	57.5 (38–74)	30.5 (17–43)	*P* < 0.001
Female gender/total (%)	19/33 (57.58%)	25/38 (65.79%)	NS
*Comorbidity *			
Diabetes mellitus	2/33 (6.06%)	0	NS
Chronic lung disease*	5/33 (15.15%)	8/38 (21.05%)	NS
Chronic cardiovascular disease	7/33 (21.21%)	1/38 (2.63%)	*P* < 0.05
Immunosuppression**	12/33 (36.36%)	11/38 (28.95%)	NS
Chronic renal disease	3/33 (9.09%)	4/38 (10.53%)	NS
Total number of patients with comorbidity	26/33 (78.79%)	19/38 (50.0%)	*P* < 0.05
*Hospitalisation *			
Time between first symptoms to hospital admission (days)	3	4	ND
Hospital admission	30/33 (90.90%)	24/38 (63.16%)	*P* = 0.01
ICU admission	2/33 (6.06%)	11/38 (28.95%)	*P* < 0.05
Length of hospital stay including ICU (days)	8.5	13.5	*P* = 0.06
*Antimicrobial treatment *			
Antiviral treatment	10/30 (30.30%)	18/38 (47.37%)	NS
Antibacterial treatment	26/33 (78.79%)	25/38 (65.79%)	NS
*Mortality *			
Overall mortality	12/33 (36.36%)	3/38 (7.89%)	*P* = 0.01
Mortality within 4 weeks of influenza diagnosis	2/33 (6.06%)	1/38 (2.63%)	NS
Mortality due to influenza complications	4/33 (12.12%)	3/38 (7.89%)	NS

ND: not determined; NS: not significant; *asthma or COPD, cystic fibrosis; **kidney/liver transplantation, solid tumor, chemotherapy, acute myelogenous leukaemia (AML), chronic myelogenous leukaemia (CML), autologous stem cell transplant (ASCT), and chronic lymphocytic leukaemia (CLL).
